# 我如何用粪菌移植治疗肠道急性移植物抗宿主病

**DOI:** 10.3760/cma.j.issn.0253-2727.2022.05.003

**Published:** 2022-05

**Authors:** 晓文 唐, 德沛 吴

**Affiliations:** 苏州大学附属第一医院血液内科，国家血液系统疾病临床医学研究中心，江苏省血液研究所，血液学协同创新中心，苏州大学造血干细胞移植研究所，苏州 215006 Department of Hematology, The First Affiliated Hospital of Soochow University, Jiangsu Institute of Hematology, National Clinical Research Center for Blood Diseases, Collaborative Innovation Center of Hematology, Institute of Hematopoietic Stem Cell Transplantation, Soochow University, Suzhou 215006, China

异基因造血干细胞移植（allo-HSCT）是多种血液系统疾病无可取代的治疗手段。移植物抗宿主病（GVHD）是其常见的并发症，也是制约患者生存的重要原因。国内资料显示，中度和重度急性GVHD（aGVHD）的发生率为13％～47％，仅有25％～30％的Ⅲ级和1％～2％的Ⅳ级aGVHD患者获得长期存活（>2年）[1]。aGVHD主要累及皮肤（81％）、胃肠道（54％）和肝脏（50％）三个器官[2]，其中胃肠道aGVHD（GI-aGVHD）是allo-HSCT后非复发死亡的主要原因。近年，利用粪菌移植（fecal microbiota transplantation, FMT）治疗aGVHD已成为研究热点。

一、典型病例

患者，男，35岁，诊断为“骨髓增生异常综合征伴多系血细胞发育异常（MDS-MLD），IPSS-R中危”，长期输血依赖。患者及家属有强烈移植意愿，但患者无HLA相合同胞和无关供者，综合考虑后选择行表兄供表弟（旁系）单倍型造血干细胞移植。患者造血重建顺利，转入移植后病房继续治疗。移植后28 d（+28 d）出现皮肤aGVHD，+33 d发生肠道aGVHD，起初患者表现为黄色稀水便，每日总量570 ml，每日腹泻次数≥7次，根据MAGIC分级标准评估患者为Ⅲ级肠道aGVHD，后患者粪便性状转为墨绿色水样便，每日总量1 070 ml，腹泻次数≥10次，并伴随出现腹痛、血便（进展为Ⅳ级肠道aGVHD）。在整个治疗期间，患者先后应用甲泼尼龙（2 mg·kg^−1^·d^−1^）、他克莫司、巴利昔单抗、重组人Ⅱ型肿瘤坏死因子受体-抗体融合蛋白、布地奈德等，均治疗无效。与患者沟通后，予放置胃空肠管行FMT，首次FMT后第2天，患者腹痛、腹泻症状没有任何改善，腹泻量反而增加（最多达到1 255 ml），但在FMT后第3天，患者腹痛程度减轻，腹泻量明显减少，最少时24 h腹泻量仅225 ml，血便渐止，1周后行第二次FMT，患者的肠道aGVHD逐步控制好转，直至完全缓解，最终患者在糖皮质激素等药物减量后病情无反复，顺利出院。

二、GI-aGVHD的发生、发展及其与肠道菌群失调的相关性

GI-aGVHD主要是由于胃肠道免疫系统稳态机制紊乱和供者细胞毒性淋巴细胞过度反应所致。预处理及感染造成上皮细胞损伤和炎症环境，患者抗原呈递细胞（APC）黏附分子、共刺激分子和抗原表达增加，呈递抗原导致供者T细胞激活、增殖、产生Th1型细胞因子、细胞毒性T细胞和先天性免疫细胞，作用于胃肠道，其肠上皮干细胞、Paneth细胞、杯状细胞、黏液层是GI-aGVHD的攻击靶点，导致肠道通透性增加、肠道紧密连接破坏、黏液层和抗菌物质减少，随后激活免疫反应受体和细胞因子风暴[3]。

健康人群中肠道菌群以拟杆菌门、厚壁菌门和放线菌门为主，占90％以上[4]。肠道菌群失调已被证实是GI-aGVHD的影响因素。allo-HSCT后，患者共生菌群向肠球菌相对转移，这种转变在随后发展为GVHD的患者中尤为明显，发生GVHD的患者的肠球菌比例由21％增加到46％，在GVHD活跃期可高达74％[5]。研究表明，aGVHD患者较非GVHD患者厚壁菌门（毛螺菌科和瘤胃球菌科）和肠球菌属占比更高，其中肠道aGVHD患者粪便中拟杆菌门占比更低。对55例allo-HSCT患者的随机对照研究中（23例接受自体FMT治疗，18例对照组），肠道微生物群组成变化的分析表明，治疗组患者重建了重要的共生群（毛螺菌科、瘤胃球菌科和拟杆菌）[6]。在严重GI-aGVHD患者中，肠道短链脂肪酸（SFCA）水平显著下降（醋酸盐75.8％，丙酸盐95.8％，丁酸盐94.6％），SCFA变化与对应的产丁酸盐菌种的增减一致[7]。布劳特氏菌（Blautia）是占优势的人类结肠毛螺菌科，具有产生丁酸盐的能力，allo-HSCT后12 d布劳特氏菌的存在与较低的复发率、GVHD死亡率和更好的总生存率有关[8]。在小鼠GI-aGVHD模型中，给予丁酸盐或产生丁酸盐的细菌（如梭状芽孢杆菌）可减轻GI-aGVHD[9]。此外，移植后粒细胞缺乏合并发热时使用具有抗厌氧共生菌活性的广谱抗生素（如亚胺培南/西司他丁、哌拉西林他唑巴坦）与GVHD相关死亡率增加有关[10]。最佳剂量他克莫司可诱导大鼠肝移植后产生适宜的免疫抑制，使移植物功能正常、肠道菌群稳定，致使包括普拉梭菌和双歧杆菌的益生菌增加，拟杆菌、肠杆菌等有害菌减少[11]。因此，特定的微生物群和代谢改变与GI-aGVHD的发生及严重程度相关。

总之，造血干细胞移植后，抗生素和免疫抑制剂的使用都会影响患者肠屏障和肠道菌群的多样性和构成变化，这些变化贯穿GI-aGVHD发生发展的全过程，同时肠道菌群失调进一步导致患者继发性炎症损害。虽然难以明确肠道菌群与GI-aGVHD的因果关系，但是可以肯定GI-aGVHD合并严重肠道菌群失调这一客观存在，提示重建肠道菌群对治疗GI-aGVHD的重要性。同时，由于GI-aGVHD以典型广泛肠黏膜屏障损害为特征表现，也提示重建肠道菌群过程中需要重视其安全性。

三、FMT用于GI-aGVHD的治疗

FMT是将健康人粪便的功能菌群移植到患者肠道内。FMT至少可追溯到公元4世纪的中国东晋时期，葛洪所著《肘后备急方》记载治疗食物中毒和严重腹泻的方法：“饮粪汁一升，即活”。2013年，FMT被纳入美国医学指南，推荐用于复发性/难治性难辨梭状芽孢杆菌感染[12]。然而，将大量微生物群系输入免疫抑制状态且合并肠黏膜屏障损坏患者的肠道内能否导致继发性微生物感染，一直是医学界担心的问题。2014年，Kelly等[13]对99例接受FMT治疗的低免疫状态（HIV感染、器官移植、肠道肿瘤、炎症性肠病使用免疫抑制剂等疾病）合并难辨梭状芽胞杆菌感染病例进行了回顾性分析，结果显示FMT的安全性可以接受。2015年，Cui等[14]研究显示，以FMT为基础升阶梯治疗方案（单次FMT为阶段1，多次FMT为阶段2，治疗无效时FMT联合糖皮质激素为阶段3）能使57.3％的糖皮质激素依赖性溃疡性结肠炎脱离糖皮质激素依赖。2016年日本Fujioka等首次应用FMT及其联合用药治疗4例糖皮质激素耐药/依赖的肠道aGVHD[15]。此后，FMT用于allo-HSCT后糖皮质激素耐药/依赖GI-aGVHD（SR-GI-aGVHD）的治疗备受关注。

目前aGVHD的一线治疗药物为糖皮质激素，GI-aGVHD的有效率为50％，其中SR-GI-aGVHD患者生存率仅为5％～30％。在一项纳入721例allo-HSCT患者的单中心回顾性研究中，Ⅱ～Ⅳ度SR-aGVHD占35.7％，进一步分析发现这部分患者胃肠道或肝脏受累风险增加≥5倍，这可能导致患者allo-HSCT后早期死亡[16]。目前FMT主要用于Ⅱ～Ⅳ度SR-GI-aGVHD，部分临床数据也证明了其疗效。2016年日本Fujioka等[15]报告：4例SR-GI-aGVHD行1～2次FMT后1周内胃肠道症状得到改善，与FMT前相比，糖皮质激素剂量平均减少69％。2017年奥地利格拉茨医科大学对于3例严重难治性肠道aGVHD（Ⅳ级）行多次FMT，均获得部分缓解（PR），其中1例患者每日腹泻量高达6 000 ml，FMT后1周内显著减少为1 000 ml [17]。2020年美国的阿姆斯特丹癌症中心的前瞻性研究：15例SR-GI-aGVHD患者接受FMT治疗，10例获得完全缓解（CR），5例为缓解（4例死亡）[17]。本中心开展了一项非随机对照的Ⅰ/Ⅱ期临床研究，共纳入41例Ⅳ度糖皮质激素耐药的肠道aGVHD患者（其中23例接受FMT治疗），治疗后21 d，FMT组CR率达56.5％（13/23），明显高于对照组的16％（3/18），随访结束时，FMT组患者的中位生存时间明显优于对照组（539 d对107 d，*P*＝0.021）[6]。以上的临床应用均采用的是鼻十二指肠方式行单次或多次FMT，且FMT过程出现的胃肠道反应均为一过性，在粪菌来源严格质控的情况下，此类患者中未见FMT相关的感染及死亡情况发生。然而，一项分析全球2000至2020年间129项研究所含的FMT相关不良事件（AE）显示，所有FMT相关严重不良事件（SAE）发生率为1.4％，均发生于肠黏膜屏障受损患者[18]。这提示需要重视FMT用于GI-aGVHD的潜在风险。

四、如何用FMT治疗肠道aGVHD

（一）FMT前准备

1. FMT来源和制备：目前用于FMT的菌种主要来源于粪菌库。2015年南京医科大学第二附属医院牵头成立我国最大粪菌库—中华粪菌库，作为公共库提供异基因来源FMT。其供者来源于6～24岁健康人群，通过完成初筛到实验室筛查和监查筛查等环节，对传染病、免疫性疾病以及遗传风险等风险因素进行排除后保留的人群为合格供者。2014年，随着世界上第一套智能粪菌分离系统GenFMTer进入实验室应用，FMT的发展进入了新阶段，基于智能化粪菌分离系统及严格质控相关漂洗过程的FMT被定义为洗涤菌群移植（WMT）[19]–[20]。WMT的主要价值在于能够使传统FMT技术的不良事件发生率下降，如治疗溃疡性结肠炎后出现的发热发生率。洗涤的过程在提高FMT安全性的同时并未降低其疗效[19]。获取用于疾病治疗的WMT为低温冻存状态。WMT依旧属于FMT范畴。

2. 患者前期肠道准备：受者肠道准备通常指的是术前肠道清洁和使用抗生素，对于GI-aGVHD并非必需。对于必须要使用抗生素治疗合并疾病（如肺部感染）的患者，FMT治疗中也可以继续使用抗生素。不过，对于同时使用抗生素的患者，建议考虑增加FMT次数。

对于合并难辨梭状芽胞杆菌感染的患者，要尽量停用可能导致难辨梭状芽胞杆菌感染的抗生素，建议尽早考虑FMT。

抑制胃酸分泌药物的使用需要结合给入途径决策，若经过鼻空肠管途径移植，不需要额外使用质子泵抑制剂；若输注的菌液需经过胃，推荐移植前使用质子泵抑制剂，以降低胃酸对菌群的影响。胃肠动力药物的使用需要结合胃肠动力状态决策，若腹泻明显，可在菌液输注前给予止泻剂，如洛哌胺（通常为2～4 mg）可用于减缓肠道蠕动，增加菌群在肠道的定植时间；若肠蠕动缓慢（如肠麻痹），可考虑在输入菌液日前肌肉注射新斯的明，减少菌群在小肠内过度生长的风险。

（二）FMT治疗aGVHD的流程

1. FMT的途径选择：上消化道途径包括口服粪菌胶囊、胃镜、鼻胃镜、鼻十二指肠/空肠置管、经皮内镜胃/肠造瘘空肠置管；下消化道途径包括结肠镜、结肠置管、乙状结肠/直肠镜和经肛门保留灌肠。具体FMT途径的选择需根据综合评估结果决定，GI-aGVHD患者推荐通过胶囊、胃管或鼻空肠管等上消化道途径。尚无FMT途径对GI-aGVHD疗效影响的直接证据。

2. FMT剂量及频次：①管道法：在近端空肠或十二指肠放置鼻十二指肠/空肠管或胃管输注菌液。WMT实现菌群富集和定量，依据洗涤菌群移植共识[20]，我们采用WMT治疗GI-aGVHD使用剂量为1～5 U/次（1 U含1.0×10^13^个细菌），每日1次，间隔时间可超过1日，患者治疗多数为1～2次。依据年龄、体重决定治疗剂量，如果低体重和低龄，所用剂量最少可为1 U。国外报道的基于实验室手工制备的方法与本中心不同，以粪便重量或粪水体积为计量单位，比如，日本采用中位粪量为126（34～307）g，与生理盐水混合成粪悬液180～230 ml，通过鼻十二指肠管4～8 min内注入[15]。其他中心采用收集新鲜供体粪便（>50 g），用无菌生理盐水稀释，获得300～500 ml的粪便悬液，并于6 h内通过鼻十二指肠管缓慢注入（10 cm^3^/min）[17]。②胶囊法：在病情允许的情况下，FMT移植前48 h停用广谱抗生素。治疗前4 h和治疗后1 h禁食，嘱患者勿压碎、咀嚼或溶解胶囊。成人移植粪便量需≥30 g，30粒胶囊中位粪便微生物含量为38.6（24.0～56.7）g，如果患者可耐受的吞服剂量为80％或更高，则认为FMT是可行的[21]–[22]。每天口服至少15粒冻干粉胶囊，粪菌胶囊的服用缺乏指南或参考标准，暂不作为急重症GI-aGVHD首选FMT方案。

移植次数和周期尚无统一标准，通常参考FMT升阶治疗策略（step-up FMT strategy）：阶段1（单次FMT）；若1周左右患者症状未改善或持续加重，则采取阶段2（≥2次FMT），评估时间仍为1周左右；阶段3：在阶段1或阶段2无效时，在FMT基础上联合常规药物治疗（如糖皮质激素、环孢素A、抗TNF-α抗体、全肠内营养等），从而发挥比单独使用FMT或传统药物都更优的疗效[23]。对于allo-HSCT后的大部分SR-GI-aGVHD患者，临床研究中使用单次FMT居多，1周后评估疗效，对于疗效不佳的患者考虑可更换新的供体菌重再行FMT治疗，如仍然无效，则应重新鉴别腹泻原因或停用FMT。该治疗策略也适用于GI-aGVHD合并难治性难辨梭状芽胞杆菌感染或者多药耐药患者，在重复FMT治疗基础上联合抗生素治疗。

（三）FMT后的监测

1. 胃肠道aGVHD的病理改变及分级标准：GI-aGVHD的患者内镜下可见胃肠道黏膜红斑水肿、出血、溃疡。显微镜下可见隐窝细胞凋亡、消失，黏膜正常结构改变[24]。目前GI-aGVHD内镜分级标准采用“Freiburg Criteria”[25]：1级：无明确标准；2级：斑点红斑；3级：透明病变；4级：融合性缺损、溃疡、黏膜脱落。再进一步对GI-aGVHD的组织行显微镜下观察分级：1级：隐窝细胞凋亡；2级：单个隐窝丢失或伴脓肿；3级：连续的隐窝坏死；4级：黏膜区域全剥除。而临床上最常采用的是改良Glucksberg标准（Ⅰ～Ⅳ级）：Ⅰ级：每日腹泻量>500 ml；Ⅱ级：每日腹泻量>1 000 ml；Ⅲ级：每日腹泻量>1 500 ml；Ⅳ级：大量腹泻伴血便或腹痛，肠梗阻。近年来更多中心采用MAGIC分级标准[26]，其中成人标准为：0级：每日腹泻<500 ml或<3次；1级：每日腹泻500～999 ml或3～4次；2级：每日腹泻1 000～1 500 ml或5～7次；3级：每日腹泻>1 500 ml或>7次；4级：严重腹痛伴或不伴肠梗阻或便血（无论排便量如何）。当然实际aGVHD的严重程度分度标准采用皮肤、胃肠道和肝脏aGVHD综合评估，才能更好地指导临床治疗。

2. 疗效评估：FMT治疗aGVHD疗效判定尚无统一标准。目前部分中心对aGVHD的完全缓解定义为供体FMT后28 d内aGVHD症状的完全消失，若在第28天开始逐渐减少免疫抑制药物治疗，患者逐渐出现症状则定义为缓解后复发。改善定义为至少有一个aGVHD分级有部分反应，如果aGVHD分级没有改善或FMT后1个月至少有1级进展，则被归类为治疗无应答[17]。根据患者在治疗后2周内腹痛、腹泻（频率和量）、血脓便等症状的严重程度，评估FMT治疗肠道aGVHD的疗效和安全性。如果腹泻和肠痉挛和（或）出血消失，或大便量在3 d内平均减少500 ml/d以上，则定义为临床缓解。如果粪便量减少<500 ml/d并且肠脓毒症和出血减轻，则定义为临床改善[6],[27]。根据FMT和随访期间的不良事件评估安全性，患者FMT后随访时间至少为8周[28]。

3. 不良事件监测：FMT相关不良事件分为短期（<1个月）和长期（≥1个月）不良事件[18]。目前，FMT被认为是一种总体上安全的治疗方法，特别是WMT显示更低的不良事件发生率，不良反应主要表现为消化道症状，为短期不良事件，且主要发生在治疗后3～6 h内，一般不需要药物处理。发热、感染是要特别重视的不良事件[19]–[30]。国外已有FMT引起多重耐药菌的传播及菌血症和败血病的个案报道。

五、总结

GVHD是制约造血干细胞移植成功的瓶颈之一，FMT是肠道aGVHD的一种新型治疗手段，具有较好的临床疗效和安全性。因其作用机制、制品的复杂性以及长期随访数据的缺乏，给临床应用及监管带来了挑战，需要我们进一步研究FMT的作用机制、改进粪便材料的制备和给药方式、确定适当的随访时间以及在复杂的监管问题上取得进展，最终改善SR-GI-aGVHD的疗效。
